# LncRNA HIF1A-AS2 promotes osteosarcoma progression by acting as a sponge of miR-129-5p

**DOI:** 10.18632/aging.102448

**Published:** 2019-12-22

**Authors:** Xuesong Wang, Lei Peng, Xiaojin Gong, Xiugong Zhang, Ruifu Sun

**Affiliations:** 1 No.1 Spinal Department of No.2 Affiliated Hospital of Qingdao University, Qingdao Central Hospital, Shandong, China; 2 Library of No.2 Affiliated Hospital of Qingdao University, Qingdao Central Hospital, Shandong, China

**Keywords:** osteosarcoma, lncRNA, HIF1A-AS2, miR-129-5p

## Abstract

Increasing studies have demonstrated that long noncoding RNAs (lncRNAs) play vital roles in tumor development and progression. However, the relationship between osteosarcoma and HIF1AAS2 remains unknown. The expression of HIF1AAS2 and miR-129-5p was detected in osteosarcoma cell lines and samples via qRT-PCR. Cell Counting Kit-8 (CCK-8) and invasion assays were performed to determine cell proliferation and invasion ability, and a dual luciferase reporter assay was performed to determine the interaction between HIF1AAS2 and miR-129-5p. We showed that the expression of HIF1A-AS2 was upregulated in the osteosarcoma samples compared with the expression in noncancerous samples. Moreover, patients with high HIF1A-AS2 expression had a shorter overall survival. Ectopic expression of HIF1A-AS2 enhanced osteosarcoma cell proliferation, cell cycle progression and invasion. We found that overexpression of miR-129-5p decreased the luciferase activity of wild-type (WT) HIF1A-AS2 but not mutant HIF1A-AS2. Ectopic expression of HIF1A-AS2 suppressed miR-129-5p expression in MG-63 cells. We demonstrated that miR-129-5p was downregulated in osteosarcoma and was negatively associated with HIF1A-AS2 expression. Furthermore, ectopic expression of miR-129-5p suppressed osteosarcoma cell proliferation, cell cycle progression and invasion. In addition, overexpression of HIF1A-AS2 promoted cell proliferation, cell cycle progression and invasion of osteosarcoma cells through the modulation of miR-129-5p. These results indicated that HIF1A-AS2 might be a potential therapeutic target for osteosarcoma.

## INTRODUCTION

Osteosarcoma, the major cause of tumor-related death in adolescents and children, is the most common malignant primary bone cancer [1–5]. Osteosarcoma is destructive and has high metastatic potential, mostly to the lungs [3, 6–8]. Despite treatment strategies that have been rapidly developed, the five-year survival rate of osteosarcoma is still unsatisfactory [9–11]. Until now, the molecular mechanism underlying the progression and development of osteosarcoma remained unknown [12–14]. Therefore, it is urgently necessary to identify new therapeutic factors or targets for osteosarcoma.

Long noncoding RNAs (lncRNAs) are a group of transcripts that lack protein-coding potential and that are longer than 200 nucleotides [15–18]. Increasing studies have implicated dysregulated lncRNAs in multiple tumors, such as hepatocellular carcinoma, lung cancer, colorectal cancer, bladder cancer and osteosarcoma [19–25]. LncRNAs play vital roles in tumor apoptosis, proliferation, differentiation, metastasis and invasion [26–30]. Recently, a new lncRNA, HIF1A-AS2, was found to be dysregulated in several tumors, such as colorectal cancer, breast cancer, glioblastoma, bladder cancer and gastric cancer [31–35]. It was found that HIF1A-AS2 promoted cell proliferation and invasion. However, the expression and potential role of HIF1A-AS2 in the development of osteosarcoma remain unknown.

In this study, we found that the expression of HIF1A-AS2 was upregulated in the osteosarcoma samples compared with the expression levels in noncancerous samples. Moreover, patients with high HIF1A-AS2 expression had a shorter overall survival than the low HIF1A-AS2 expression group. Furthermore, we studied the function of HIF1A-AS2 in osteosarcoma cells and indicated that ectopic expression of HIF1A-AS2 enhanced osteosarcoma cell proliferation, cell cycle progression and invasion.

## RESULTS

### HIF1A-AS2 was upregulated in osteosarcoma and was related to poor survival

**Figure 1:**
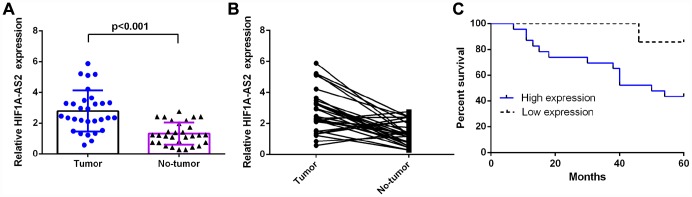
**HIF1A-AS2 was upregulated in osteosarcoma and was related to poor survival.** (**A**) The expression of HIF1A-AS2 in 30 osteosarcoma samples and their noncancerous pairs was detected by qRT-PCR. U6 was used as the internal control. (**B**) The expression of HIF1A-AS2 was upregulated in 23 cases (23/30; 77 %) compared to the expression in adjacent tissues. (Defined as a cutoff of Log 2.0-fold-change >1) (**C**) The high HIF1A-AS2 expression group had a shorter overall survival than the low HIF1A-AS2 expression group (median overall survival =50 vs. 92 months, respectively; log-rank p<0.01).

RT-qPCR was performed to measure HIF1A-AS2 expression in 30 osteosarcoma samples and paired adjacent normal tissue, which were normalized to U6. The expression of HIF1A-AS2 was significantly higher in the osteosarcoma samples compared to the expression levels in the paired normal controls (Figure 1A, mean expression in the osteosarcoma samples versus the normal tissue = 2.790 ± 0.2451 vs. 1.321 ± 0.1329, respectively; p<0.001). In addition, the expression of HIF1A-AS2 was upregulated (defined as a cutoff set at log 2.0-fold-change >1) in 23 cases (23/30; 77 %) compared to the expression in adjacent normal tissues (Figure 1B). Moreover, we showed that the median survival of osteosarcoma patients with high HIF1AAS2 expression in primary tumors was 50 months, which was shorter than those with low HIF1AAS2 expression (92 months) (Figure 1C, median overall survival, =50 vs. 92 months; log-rank p<0.01).

### HIF1A-AS2 promoted osteosarcoma cell proliferation, cell cycle progression and invasion

**Figure 2:**
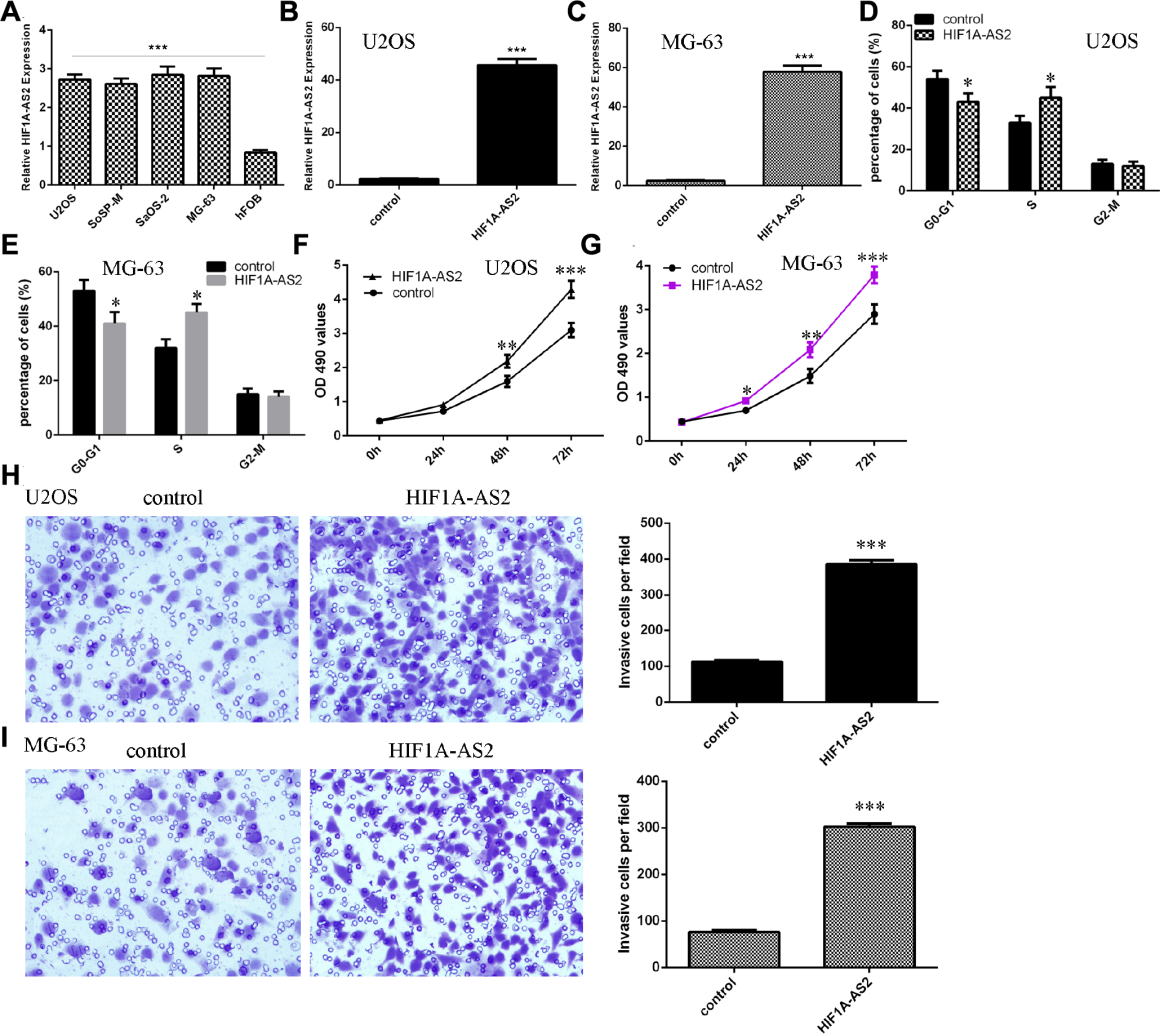
**HIF1A-AS2 promoted osteosarcoma cell proliferation, cell cycle progression and invasion.** (**A**) The expression of HIF1A-AS2 in osteosarcoma cell lines (U2OS, SoSP-M, SaOS-2, MG-63) and an osteoblast cell line (hFOB) was measured by qRT-PCR. (**B**) The expression of HIF1A-AS2 in U2OS cells was determined by qRT-PCR. (**C**) The expression of HIF1A-AS2 in MG-63 cells was determined by qRT-PCR. (**D**) Ectopic expression of HIF1A-AS2 increased the S phase of U2OS cells. (**E**) Ectopic expression of HIF1A-AS2 promoted the S phase of MG-63 cells. (**F**) Overexpression of HIF1A-AS2 promoted U2OS cell proliferation. (**G**) Ectopic expression of HIF1A-AS2 promoted MG-63 cell growth. (**H**) Ectopic expression of HIF1A-AS2 increased U2OS cell invasion, and the relative invasive cells are shown. (**I**) Ectopic expression of HIF1A-AS2 increased MG-63 cell invasion, and the relative invasive cells are shown. *p<0.05, **p<0.01 and ***p<0.001.

Next, we showed that the expression of HIF1A-AS2 was upregulated in osteosarcoma cell lines (U2OS, SoSP-M, SaOS-2, MG-63) compared to the expression in the osteoblast cell line (hFOB) (Figure 2A, p<0.001). Then, we confirmed that pcDNA-HIF1A-AS2 could enhance the expression of HIF1A-AS2 in U2OS (Figure 2B, p<0.001) and MG-63 (Figure 2C, p<0.001) cells by using qRT-PCR analysis. Moreover, ectopic expression of HIF1A-AS2 increased the S phase of the U2OS (Figure 2D, p<0.05) and MG-63 (Figure 2E, p<0.05) cells compared to that of the control group. The MTT analysis was conducted to measure the growth of the U2OS and MG-63 cells after ectopic expression of HIF1A-AS2. Overexpression of HIF1A-AS2 promoted U2OS (Figure 2F) and MG-63 (Figure 2G) cell growth. Ectopic expression of HIF1A-AS2 increased U2OS (Figure 2H, p<0.001) and MG-63 (Figure 2I, p<0.001) cell invasion, and the relative invasive cells are shown.

### Ectopic expression of HIF1A-AS2 inhibited miR-129-5p expression

**Figure 3:**
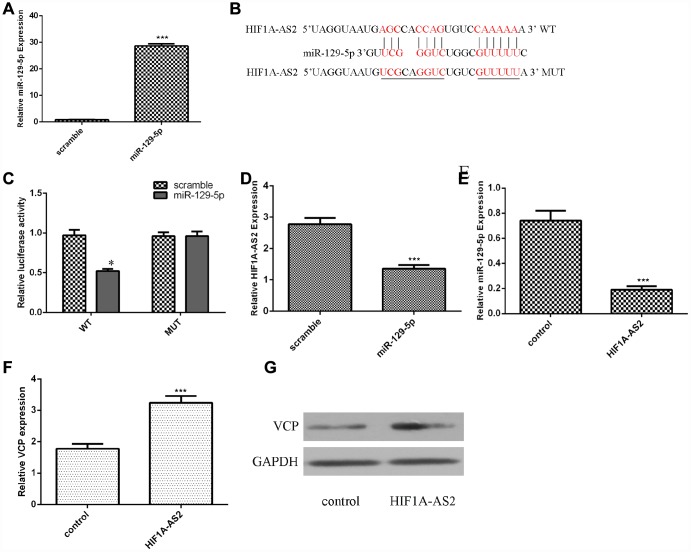
**Ectopic expression of HIF1A-AS2 inhibited miR-129-5p expression.** (**A**) The expression of miR-129-5p in MG-63 cells was detected by using qRT-PCR analysis. (**B**) The binding sites between HIF1A-AS2 and miR-129-5p obtained from bioinformatics analysis are shown. (**C**) We showed that overexpression of miR-129-5p can decrease the luciferase activity of wild-type (WT) HIF1A-AS2 but not mutant HIF1A-AS2. (**D**) Overexpression of miR-129-5p decreased the expression of HIF1A-AS2 in MG-63 cells. (**E**) Ectopic expression of HIF1A-AS2 suppressed miR-129-5p expression in MG-63 cells. (**F**) Overexpression of HIF1A-AS2 increased VCP expression in MG-63 cells. (**G**) The protein expression of VCP was detected by Western blot. GAPDH was used as the control. *p<0.05.

Using qRT-PCR analysis, we confirmed that the miR-129-5p mimic could enhance the expression of miR-129-5p in MG-63 cells (Figure 3A, p<0.001). The binding sites between HIF1A-AS2 and miR-129-5p were identified by using bioinformatics analysis (Figure 3B). A luciferase reporter assay was conducted to validate the binding site combinations. We showed that overexpression of miR-129-5p could decrease the luciferase activity of wild-type (WT) HIF1A-AS2 but not that of the mutant HIF1A-AS2 (Figure 3C, p<0.05). Overexpression of miR-129-5p decreased the expression of HIF1A-AS2 in MG-63 cells (Figure 3D, p<0.01). Ectopic expression of HIF1A-AS2 suppressed miR-129-5p expression in MG-63 cells (Figure 3E, p<0.01). Furthermore, we showed that overexpression of HIF1A-AS2 increased VCP expression in MG-63 cells (Figure 3F, p<0.01). We also confirmed that ectopic expression of HIF1A-AS2 promoted VCP protein expression in MG-63 cells (Figure 3G).

### MiR-129-5p was downregulated in osteosarcoma and was negatively correlated with HIF1A-AS2 expression

**Figure 4:**
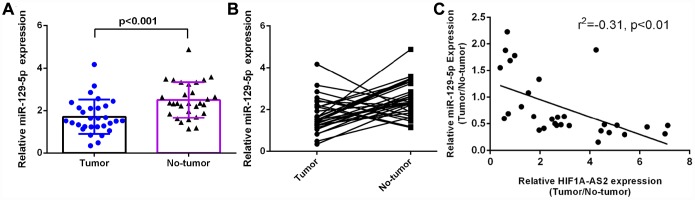
**miR-129-5p was downregulated in osteosarcoma and was negatively related to HIF1A-AS2 expression.** (**A**) The miR-129-5p expression in 30 osteosarcoma samples and their noncancerous pairs was determined by qRT-PCR. U6 was used as the internal control. (**B**) The expression of miR-129-5p was downregulated in 22 cancerous tissues (22/30; 73%) compared to the adjacent tissues. (Defined as a cutoff of Log 2.0-fold-change >1) (**C**) miR-129-5p expression was negatively correlated with HIF1A-AS2 expression in osteosarcoma samples by using Pearson's correlation coefficient analysis.

RT-qPCR was performed to analyze miR-129-5p expression in 30 osteosarcoma samples and paired adjacent normal tissues, which were normalized to U6. The expression of miR-129-5p was significantly lower in the osteosarcoma samples than in the paired normal control tissues (Figure 4A, mean expression in the osteosarcoma tissue versus the normal tissue =1.710 ± 0.1479 vs. 2.496 ± 0.1523, respectively; p<0.001). In addition, the expression of miR-129-5p was downregulated (defined as a cutoff set at Log 2.0-fold-change >1) in 22 cases (22/30; 73%) compared to the expression in adjacent normal tissues (Figure 4B). Moreover, we showed that miR-129-5p expression was negatively correlated with HIF1A-AS2 expression (Figure 4C, r^2^=-0.31, p<0.01) by using Pearson's correlation coefficient analysis.

### miR-129-5p suppressed osteosarcoma cell proliferation, cell cycle progression and invasion

**Figure 5:**
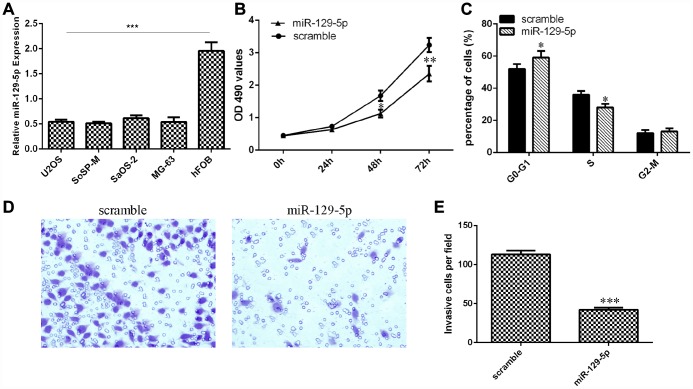
**miR-129-5p suppressed osteosarcoma cell proliferation, cell cycle progression and invasion.** (**A**) The expression of miR-129-5p in osteosarcoma cell lines (U2OS, SoSP-M, SaOS-2, MG-63) and an osteoblast cell line (hFOB) was measured by qRT-PCR. (**B**) Overexpression of miR-129-5p suppressed MG-63 cell growth. (**C**) Ectopic expression of miR-129-5p decreased the S phase of MG-63 cells compared to that of the scramble group. (**D**) Ectopic expression of miR-129-5p decreased MG-63 cell invasion. (**E**) The relative invasive cells are shown. *p<0.05, **p<0.01 and ***p<0.001.

Next, we showed that the expression of miR-129-5p was downregulated in osteosarcoma cell lines (U2OS, SoSP-M, SaOS-2, MG-63) compared to the expression in the osteoblast cell line (hFOB) (Figure 5A, p<0.001). The MTT analysis was conducted to measure the growth of MG-63 cells after ectopic expression of miR-129-5p. Overexpression of miR-129-5p suppressed MG-63 cell growth (Figure 5B). Ectopic expression of miR-129-5p decreased the S phase of the MG-63 cells compared to that of the control group (Figure 5C, p<0.05). Ectopic expression of miR-129-5p decreased MG-63 cell invasion, and the relative invasive cells are shown (Figure 5D and 5E, p<0.001).

### HIF1A-AS2 regulated cell proliferation, cell cycle progression and invasion of osteosarcoma cells through the modulation of miR-129-5p

**Figure 6:**
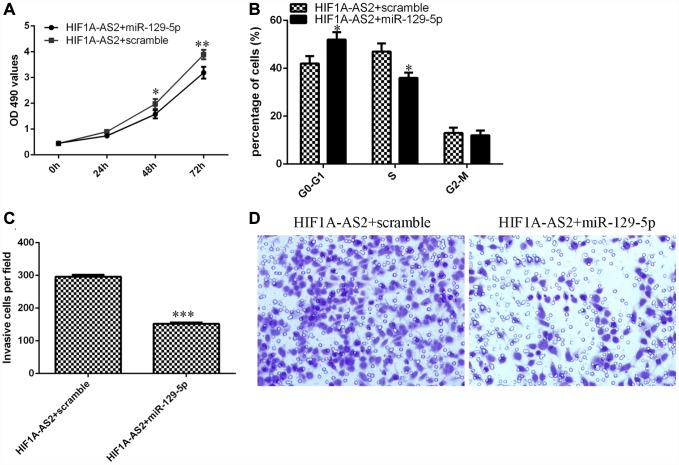
**HIF1A-AS2 regulates cell proliferation, cell cycle progression and invasion of osteosarcoma cells through the modulation of miR-129-5p.** (**A**) The cell proliferation of MG-63 cells was determined by MTT analysis. (**B**) Ectopic expression of miR-129-5p decreased the S phase of HIF1A-AS2-overexpressing MG-63 cells compared to that of the scrambled group. (**C**) Elevated expression of miR-129-5p suppressed the invasion of HIF1A-AS2-overexpressing MG-63 cells compared to the invasion of the scrambled group. (**D**) The relative invasive cells are shown. *p<0.05, **p<0.01 and ***p<0.001.

We aimed to determine whether HIF1A-AS2 acted by silencing miR-129-5p expression. The MTT assay indicated that the miR-129-5p mimic could inhibit the promotion of proliferation induced by HIF1A-AS2 overexpression (Figure 6A). Ectopic expression of miR-129-5p decreased the S phase of the HIF1A-AS2-overexpressing MG-63 cells compared to that of the scrambled group (Figure 6B, p<0.05). In addition, elevated expression of miR-129-5p suppressed the invasion of HIF1A-AS2-overexpressing MG-63 cells compared to that of the scramble group (Figure 6C and 6D, p<0.001).

## DISCUSSION

Increasing studies have suggested that lncRNAs perform critical functions in the modulation of genes that regulate cancer growth, migration, and apoptosis, which has increased our understanding of biological behaviors of several diseases, especially tumors including osteosarcoma [22, 28, 36–39]. Moreover, previous evidence has shown that lncRNAs are therapeutic targets and valuable biomarkers [40, 41]. A previous study suggested that a new lncRNA, HIF1A-AS2, located on chromosome 14q23.2 was upregulated in several cancers, such as neuroblastoma, chronic myeloid leukemia, colorectal cancer and bladder cancer [33, 35]. HIF1A-AS2 was overexpressed in gastric cancer, and HIF1A-AS2 knockdown could suppress gastric cancer cell proliferation and tumorigenesis [31]. However, the relationship between osteosarcoma and HIF1AAS2 remains unknown.

This is the first study to explore the function of HIF1A-AS2 in osteosarcoma. In our research, we detected the mean expression levels and clinical importance of HIF1A-AS2 in osteosarcoma and further investigated its cellular function in osteosarcoma cell lines. Our study demonstrated that the expression of HIF1A-AS2 was upregulated in osteosarcoma samples compared with the expression in noncancerous samples. Moreover, patients with high HIF1A-AS2 expression had a shorter overall survival than the low HIF1A-AS2 expression group. Furthermore, we studied the function of HIF1A-AS2 in osteosarcoma cells and demonstrated that ectopic expression of HIF1A-AS2 enhanced osteosarcoma cell proliferation, cell cycle progression and invasion. Suppressing HIF1A-AS2 expression may present one therapeutic strategy for curing osteosarcoma in the clinical setting. More work is required to explain the molecular mechanisms of HIF1A-AS2 in the development of osteosarcoma.

Previous studies have demonstrated that lncRNAs exert crucial functions in regulating biological cell processes by acting as ‘sponges’ for miRNAs [25, 42]. Lin et al showed that HIF1A-AS2 promoted colorectal cancer progression and epithelial-mesenchymal transition by suppressing miR-129-5p expression. To study the downstream genes of HIF1A-AS2, we used bioinformatics to find a potential miRNA with complementary binding at the HIF1A-AS2 3’-UTR. Furthermore, we performed a luciferase reporter assay and found that overexpression of miR-129-5p could decrease the luciferase activity of wild-type (WT) HIF1A-AS2 but not mutant HIF1A-AS2. Ectopic expression of HIF1A-AS2 suppressed miR-129-5p expression in MG-63 cells. Several studies have suggested that miR-129-5p plays important roles in the development of tumors such as hepatocellular carcinoma, colon cancer, breast cancer, lung cancer, gastric cancer and osteosarcoma [43–48]. Long et al [48] showed that miR-129-5p expression might be regulated by demethylation and that miR-129-5p suppressed osteosarcoma cell invasion and migration by targeting valosin-containing protein (VCP) in osteosarcoma. In addition, Liu et al [49] indicated that lncRNA MALAT1 increased osteosarcoma progression by regulating HMGB1 expression via miR-129-5p and miR-142-3p. In our study, we observed that miR-129-5p was downregulated in osteosarcoma and was negatively related to HIF1A-AS2 expression. Furthermore, ectopic expression of miR-129-5p suppressed osteosarcoma cell proliferation, cell cycle progression and invasion. In addition, overexpression of HIF1A-AS2 promoted cell proliferation, the cell cycle progression and invasion of osteosarcoma cells through the modulation of miR-129-5p.

In conclusion, the data from our study suggested that upregulated HIF1A-AS2 acted as an oncogene in osteosarcoma and induced the tumorigenesis of osteosarcoma by regulating miR-129-5p expression, indicating that HIF1A-AS2 might be a potential therapeutic target for osteosarcoma.

## MATERIALS AND METHODS

### Tissue samples

Thirty paired osteosarcoma samples and matched adjacent normal bone samples from osteosarcoma patients who underwent surgery were collected from the No. 2 Affiliated Hospital of Qingdao University. The surgically removed tissues were quickly stored in liquid nitrogen until they were used. This research was approved by the local clinical ethics committee of the No. 2 Affiliated Hospital of Qingdao University, and our study was performed following the principles of the Declaration of Helsinki. Written informed consent was obtained from all patients.

### Cell culture and transfection

Human osteosarcoma cell lines (U2OS, SoSP-M, SaOS-2, MG-63) and an osteoblast cell line (hFOB 1.19) were purchased from the American Type Culture Collection (ATCC) (Rockville, MD). These cell lines were maintained in DMEM (Dulbecco’s modified Eagle’s medium) (Invitrogen, Carlsbad, CA, USA) supplemented with FBS (fetal bovine serum) and streptomycin/penicillin. miR-129-5p mimic and scramble, pcDNA-HIF1A-AS2 and pcDNA-control were purchased from Ambion. Cells were transfected with these vectors using Lipofectamine 2000 (Invitrogen, USA) according to the manufacturer’s instructions.

### RNA isolation and real-time PCR

Total RNA from the samples or cell lines was separated using a TRIzol kit (Invitrogen, Carlsbad, USA). The expression of miRNA, lncRNA and mRNA was determined using SYBR Green (TaKaRa) with the Applied Biosystems 7900 system according to the manufacturer’s recommendations. The qRT-PCR data were normalized using the 2^-ddCt^ method. The expression of miRNA and lncRNA was normalized to U6. The expression of mRNA was normalized to GAPDH. The primer sequences of these genes were as follows: U6 forward, 5ʹ-CGCTAGCACATATCGGC TA-3ʹ and reverse, 5ʹ-TTCTGCGACGAATTTGTCAT-3ʹ; HIF1A-AS2 forward, 5ʹ-TCTGTGGCTCAGTTCCT TTTGT-3ʹ and reverse, 5ʹ-ATGTAGGAAGTGCCA GAGCC-3ʹ; GAPDH forward, 5ʹ-CGCTCTCTGCTCC TCCTGTTC-3ʹ and reverse, 5ʹ-ATCCGTTGACTCC GACCTTCAC-3ʹ.

### Cell growth and invasion assay

Cell growth was determined using MTT (3-(4,5-dimethylthiazol-2-yl)-2,5-diphenyltetrazolium bromide). Osteosarcoma cells were seeded in a 96-well plate, and 10 μL of MTT was added to each well. After three hours, the absorbance value at 490 nm was measured. For cell invasion, cells were cultured in the top of a Matrigel-coated chamber with serum-free DMEM. Medium containing FBS was added to the lower chamber. After 48 hours, cells that invaded the lower well were fixed, stained with hematoxylin and counted. For the cell cycle assay, flow cytometric analysis was used. The cells were fixed with ethanol (70 %) and incubated with RNase A. Subsequently, these cells were incubated with propidium iodide (PI) (Becton–Dickinson, CA, USA) and analyzed on the FACScan flow cytometer (San Jose, USA).

### Western blotting

Protein from cells or tissues was extracted using RIPA buffer (Pierce) in accordance with the manufacturer’s recommendations. The protein concentration was quantified using a BCA kit. Equal amounts of protein were separated with SDS–acrylamide gel and transferred into a PVDF membrane (Millipore). After blocking with nonfat milk, the membrane was incubated with anti-VCR and anti-GAPDH antibodies. The membrane was then incubated with secondary antibody at room temperature for half an hour. The signal was determined by enhanced chemiluminescence (ECL). GAPDH was used as the control.

### Luciferase reporter assay

MG-63 cells were cultured in a 24-well plate. The binding sites of miR-129-5p and the HIF1A-AS2 3’UTR and the wild-type 3’UTR were subcloned into the pGL3 luciferase promoter vector (Promega, USA). Cells were cotransfected with 3’UTR and wild-type 3’UTR HIF1A-AS2 or miR-129-5p mimics and luciferase reporter plasmids (Promega, USA) using Lipofectamine 2000 following the manufacturer’s protocol. Luciferase activity was detected using the Dual Luciferase Reporter Assay kit (Promega).

### Statistical analysis

Data are presented as the mean ± standard deviation (SD) and were measured by SPSS 17.0 (IBM, Chicago, USA). The two-tailed Student’s t test and ANOVA were performed to assess the significance differences. The overall survival analysis of these osteosarcoma patients was performed by log-rank test, and the correlation between HIF1A-AS2 or miR-129-5p was determined by Pearson's correlation coefficient analysis. P<0.05 was accepted as statistically significant.
